# Supplementary material for: Noninvasive Physical Plasma as Innovative and Tissue-Preserving Therapy for Women Positive for Cervical Intraepithelial Neoplasia
Source: Cancers (Basel). 2022 Apr 12;14(8):1933. doi: 10.3390/cancers14081933 (PMC9027888; doi:10.3390/cancers14081933)
Supplement: Supplementary file 1 [file cancers-14-01933-s001.zip › Cancers-1658345-Supplementary Materials.pdf]

cancers

MDPI

Noninvasive Physical Plasma as Innovative and Tissue-Preserving Therapy for Women Positive for Cervical Intraepithelial Neoplasia

Julia Marzi, Matthias B. Stope, Melanie Henes, André Koch, Thomas Wenzel, Myriam Holl, Shannon L. Layland, Felix Neis, Hans Bösmüller, Felix Ruoff, Markus Templin, Bernhard Krämer , Annette Staebler, Jakob Barz, Daniel A. Carvajal Berrio, Markus Enderle [8], Peter M. Loskill , Sara Y. Brucker, Katja Schenke-Layland and Martin Weiss

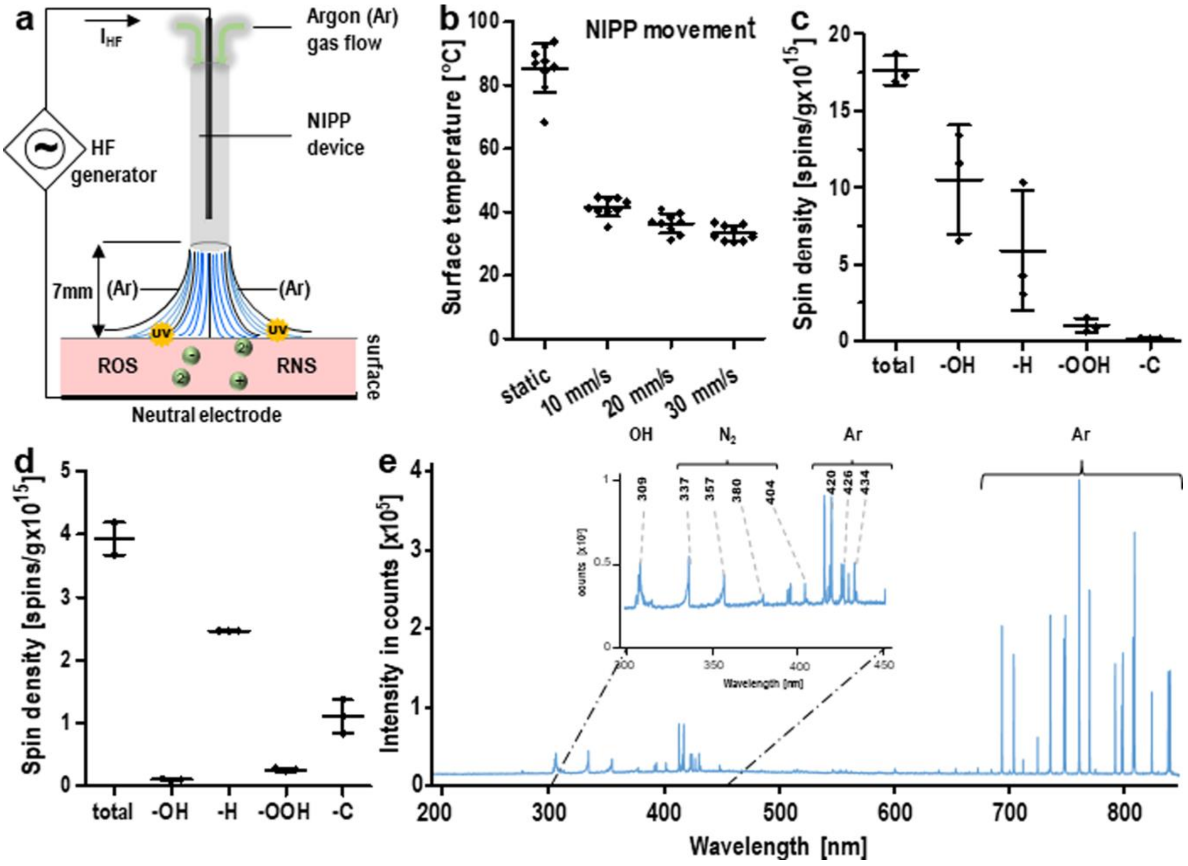

Figure S1. Physical and chemical characterization of the NIPP effluent used in tissue and patient treatment. (**a**), Schematic explaining the physicochemical principles of NIPP. Tissue application of NIPP results in the generation of biologically reactive species (ROS and RNS) as the main active NIPP components upon contact of the NIPP effluent with the ambient atmosphere and the tissue surface. HF: high-frequency. (**b**), The surface temperature in °C during static (5 s) and dynamic (mm/s) ex vivo treatment of cervical tissue (mean ± SD). (**c**,**d**), Electron spin resonance spectroscopic identification of oxygen-centered reactive species (total species, -OH, -H, -OOH, -C; mean ± SD) bound to DMPO spin traps during NIPP treatment of DPBS (c) and DPBS-soaked cervical tissue (d). (**e**), Integrated optical emission spectroscopy led to the identification of a specific peak for OH (309 nm), the nitrogen 2nd positive system $N_2(C^3\sigma_u - B^3\sigma_g)$ at 337.0 nm, 357.6 nm, 380.4 nm, 405.8 nm, and argon Ar(5p->4s) at 416.4 nm and 419.1 nm, as well as Ar(4p->3s) in various peaks from 696.5–852.1 nm.

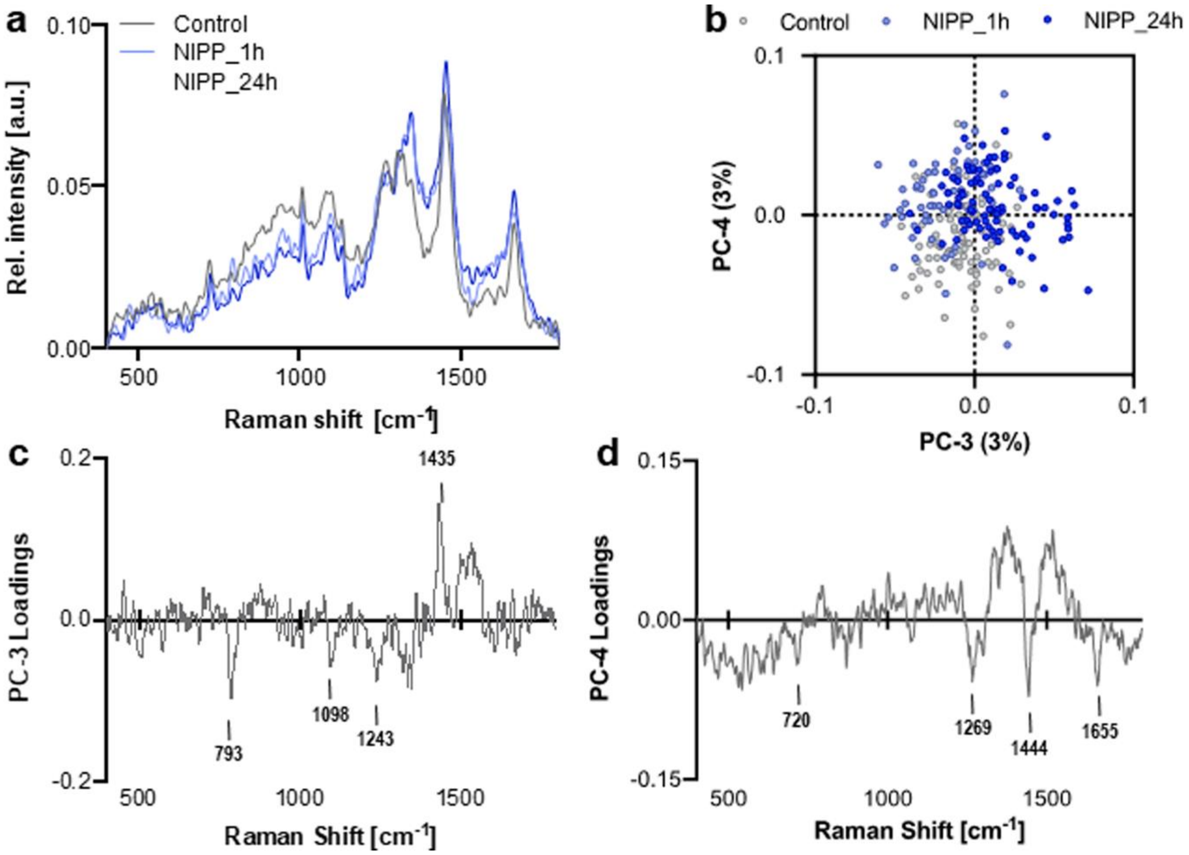

Figure S2. Raman microspectroscopy and PCA show cellular changes after NIPP treatment by noninvasive fingerprinting. (**a**), Representative single-cell Raman spectra acquired from 30 s NIPP-treated SiHa cells incubated for 1 or 24 h. (**b**), PCA demonstrated a separation in the PC-3 vs. PC-4 score plot. (**c**), Corresponding PC-3 loading plot indicating changes in DNA after NIPP treatment. (**d**), PC-4 loading plot indicating changes in phospholipid composition after NIPP treatment.

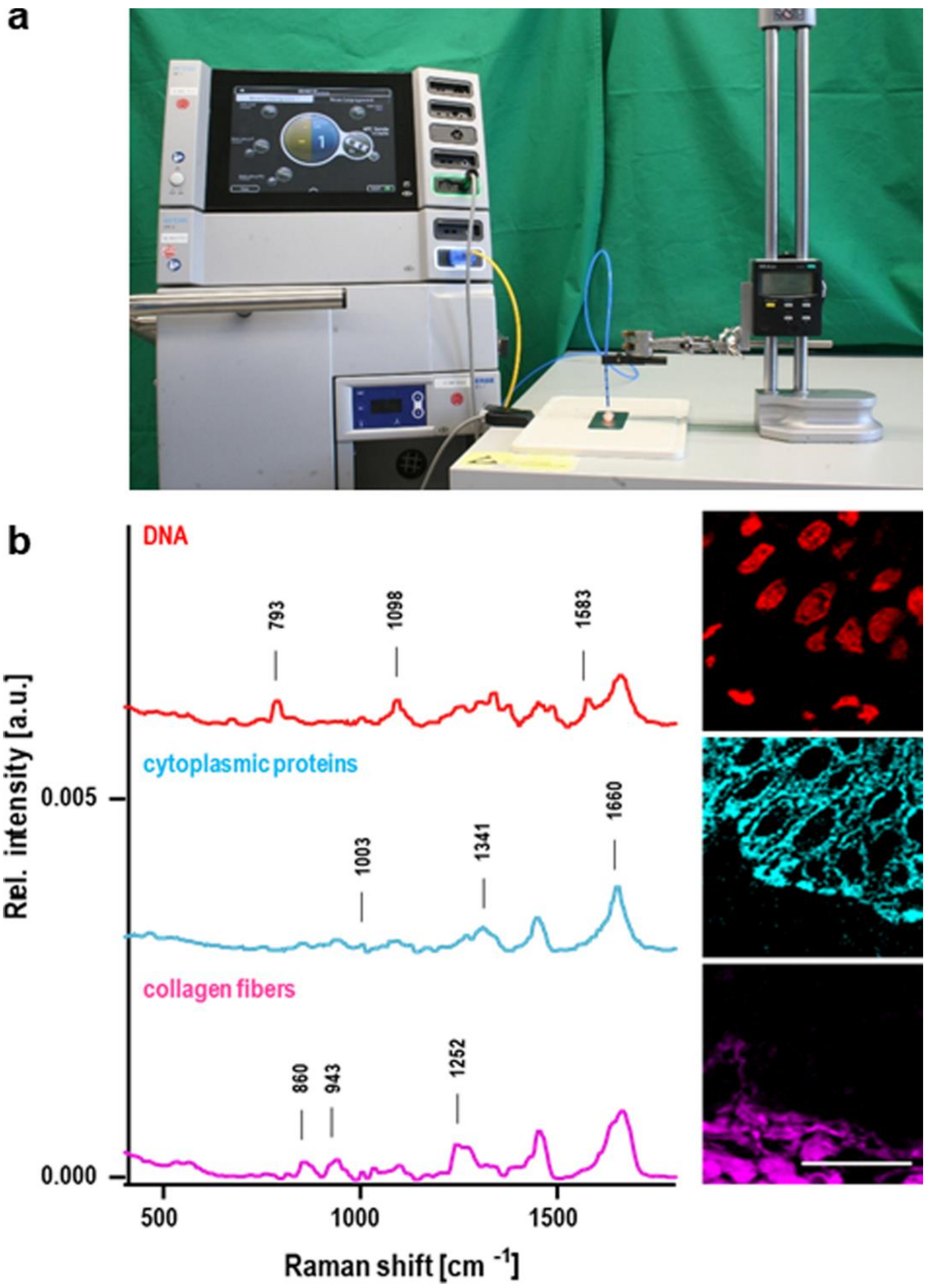

Figure S3. Tissue substructures can be visualized in a marker-independent manner by Raman imaging. (**a**), Experimental setup for ex vivo NIPP treatment of cervical tissue. (**b**), True component analysis (TCA) based on specific Raman signatures allowed us to localize nuclei (red), cytoplasmic proteins (light blue) and collagen I fibers (pink) by generating false color-coded intensity distribution heatmaps for each component in cervical tissue; the scale bar equals 50 µm.

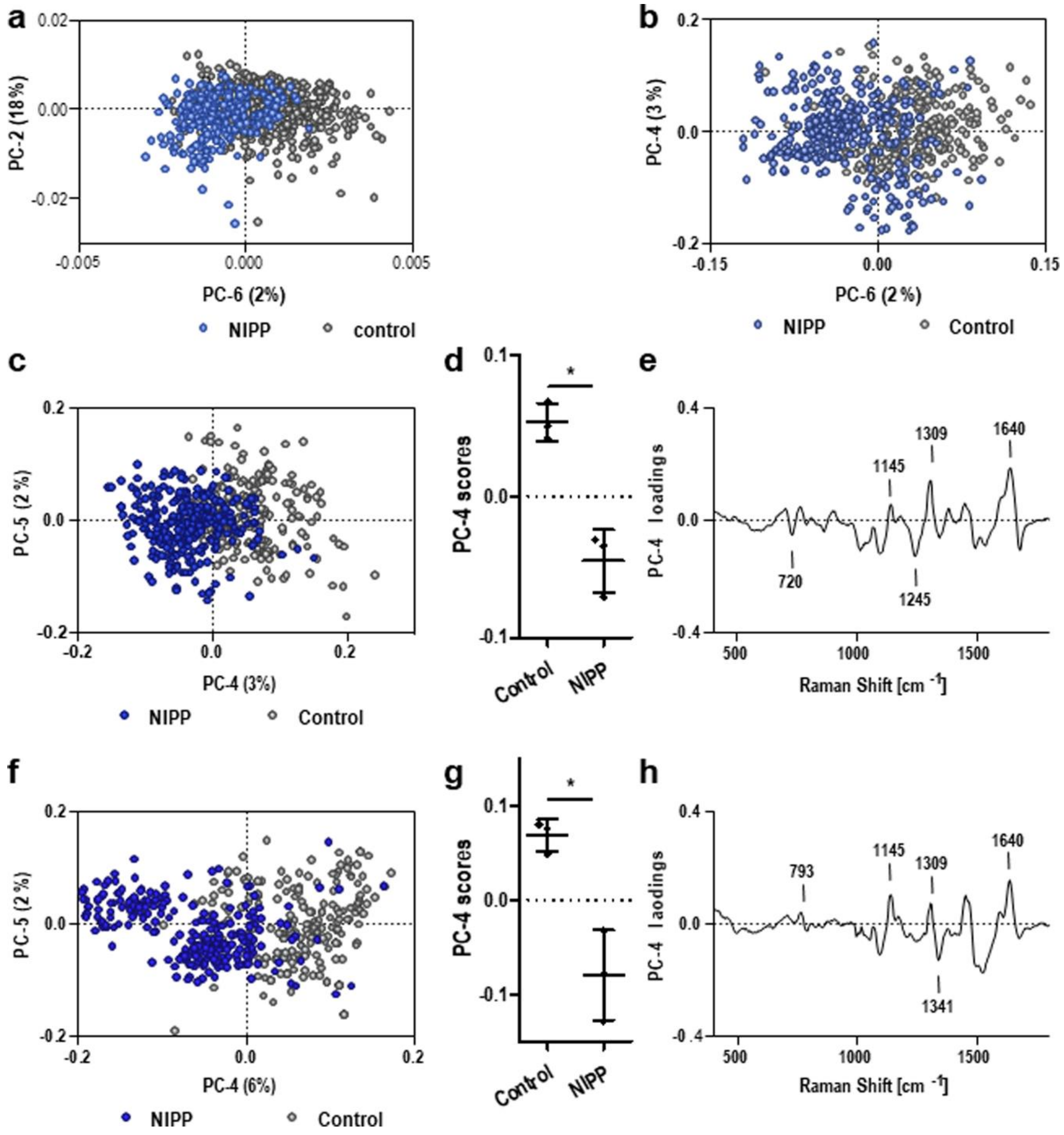

Figure S4. PCA of the nuclear features of in vivo NIPP-treated tissues demonstrates NIPP-induced cellular changes in superficial and basal tissue layers. (**a**), PCA score plots for the columnar and (**b**) squamous epithelium, as further analyzed in Figure 4. Discrimination of (**c**–**e**) superficial and (**f**,**g**) basal nuclei of the squamous epithelium and subsequent PCA analyses demonstrated a clear separation of control and NIPP-treated tissues for both tissue depths, as indicated in the score plots (**c**,**f**), and the statistical comparison of the average score values (d, g; mean ± SD; paired *t* test; * $p < 0.05$). (**e**,**h**), Corresponding spectral changes are indicated in the loading plots.

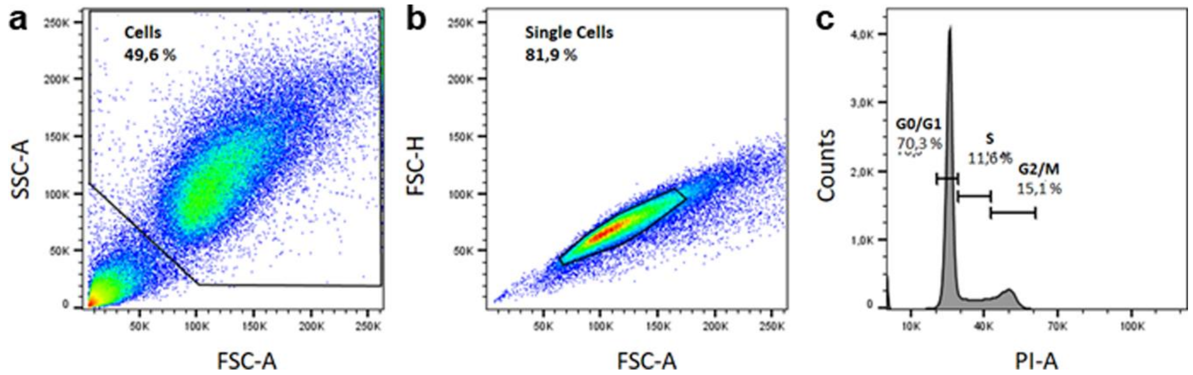

Figure S5. Gating strategy for flow cytometry. a, b, Representative flow cytometry plots showing the gating strategy. Signals were quantified using a FACSFortessa device (BD Bioscience) and FACS DIVA software v9 (BD Biosciences). Raw data were analyzed using FlowJo software v10 (FlowJo LLC). (**a**), Forward- and side-scatter (FSC-H and SSC-H) characteristics were used to exclude dead cells and cell debris. (**b**), Forward scatter area and height (FSC-A and FSC-H) characteristics were used to exclude cell doublets. (**c**), Representative DAPI histogram plot and markers used to quantitate the percentage of cells in each cell cycle phase.

Table S1. Patient characteristics for ex vivo NIPP treatment.

No. of Patients, n (%)	6 (100)
Median age, years (range)	59.0 (45–67)
Gravidity, *n* (range)	1.8 (0–3)
Parity, *n* (range)	1.5 (0–2)
Ovarian function, *n* (%)	
Premenopausal	1 (16.7)
Postmenopausal	5 (83.3)
Cause for surgery	
Hypermenorrhea	1 (16.7)
Genital descensus	5 (83.3)
Previous surgery	5 (83.3)
Conization	2 (33.3)
Uterine abrasion	3 (50.0)

Table S2. Raman shifts and their molecular assignments.

Raman Shift [cm^{-1}]	Molecular Assignment	Structure	References
714–720	N-CH_3 stretching (choline)	Membrane lipids	[37,38]
766	N-H bending (tryptophane)	Proteins	[39]
793	Ring breathing of DNA bases (U, T, C)	DNA	[40,41]
860	Proline	Collagen	[42]
943	C-C skeletal backbone	Collagen	[42]
1003–1009	C-C ring bending	Phenylalanine (proteins)	[39]
1018	Cytosine, adenine	DNA	[43]
1098	Phosphate (PO_2) backbone	DNA	[37,38]
1130–1133	C=O acyl chains (fatty acids)	Lipids	[37,38]
1145	C-C stretch	DNA	[44]
1243	Cytosine, adenine	DNA	[41]
1252	Amide III	Proteins / collagen	[42]
1269	Fatty acids, unsaturated	Lipids (cell membrane)	[37,38]
1301–1309	CH_3/CH_2 twisting	Lipids	[37,38]
1341	CH deformation	Proteins	[39]
1380	CH_3 deformation	Methylation (DNA)	[45,46]
1435–1440	C-H deformation	Lipids (cell membrane)	[37,38]
1474	Guanine	DNA	[46,47]
1580–1583	Pyrimidine breathing (cytosine)	Methylation (DNA)	[45,46]
1592	NH_2	Cytosine, guanine, adenine (DNA)	[41]
1655	C=C, fatty acids	Cell membrane (lipids)	[37,38]
1660	Amide I	Collagen (proteins)	[42]
1678	Uracil	DNA	[41]

1696	Cytosine, thymine	DNA	[43]

Table S3. Patient characteristics for in vivo NIPP treatment.

**No. of Patients, n (%)**	**6 (100)**
Median age, years (range)	29.6 (21–37)
Gravidity, *n* (range)	0.75 (0–2)
Parity, *n* (range)	0.75 (0–2)
Histology, n (%)	
LSIL (CIN1)	3 (50)
HSIL (CIN2)	3 (50)
